# Evidence of Latitudinal Migration in Tri-colored Bats, *Perimyotis subflavus*

**DOI:** 10.1371/journal.pone.0031419

**Published:** 2012-02-22

**Authors:** Erin E. Fraser, Liam P. McGuire, Judith L. Eger, Fred J. Longstaffe, M. Brock Fenton

**Affiliations:** 1 Department of Biology, The University of Western Ontario, London, Ontario, Canada; 2 Department of Natural History, Royal Ontario Museum, Toronto, Ontario, Canada; 3 Department of Earth Sciences, The University of Western Ontario, London, Ontario, Canada; University of Regina, Canada

## Abstract

**Background:**

Annual movements of tri-colored bats (*Perimyotis subflavus*) are poorly understood. While this species has been considered a regional migrant, some evidence suggests that it may undertake annual latitudinal migrations, similar to other long distance North American migratory bat species.

**Methodology/Principal Findings:**

We investigated migration in *P. subflavus* by conducting stable hydrogen isotope analyses of 184 museum specimen fur samples and comparing these results (δD_fur_) to published interpolated δD values of collection site growing season precipitation (δD_precip_). Results suggest that the male molt period occurred between June 23 and October 16 and 33% of males collected during the presumed non-molt period were south of their location of fur growth. For the same time period, 16% of females were south of their location of fur growth and in general, had not travelled as far as migratory males. There were strong correlations between δD_fur_ from the presumed molt period and both growing season δD_precip_ (males – *r* ^2^ = 0.86; *p*<0.01; females – *r* ^2^ = 0.75; *p*<0.01), and latitude of collection (males – *r* ^2^ = 0.85; *p*<0.01; females – *r* ^2^ = 0.73; *p*<0.01). Most migrants were collected at the northern (>40°N; males and females) and southern (<35°N; males only) extents of the species' range.

**Conclusions/Significance:**

These results indicate a different pattern of migration for this species than previously documented, suggesting that some *P. subflavus* engage in annual latitudinal migrations and that migratory tendency varies with latitude and between sexes. We suggest that this species' hibernation ecology makes it particularly susceptible to long winters, making migration from the northern extent of the species' range to more southern hibernacula preferable for some individuals. Fur δD values for some of the northern individuals may indicate an increase in the currently accepted northern range of this species. Sex-biased differences in migration may be the result of differences in reproductive pressures.

## Introduction

Many species of North American bats migrate and employ several strategies to do so. Some species are regional migrants, radiating annually from winter hibernation sites to summer sites, then travelling among swarming sites in the autumn [1]–[4]. Bats engaging in this type of migration have been recorded travelling distances of 500 km or more [2], [5] and may move in any direction to hibernacula. Other species are latitudinal migrants, travelling south in the autumn and north in the spring [6]–[8]. There is evidence that one species of latitudinal migrant, the hoary bat (*Lasiurus cinereus*), may travel >2000 km one way during annual movements [7].

Tri-colored bats (*Perimyotis subflavus*, formerly included in genus *Pipistrellus* [9], [10]) are common in eastern North America ranging from Central America in the south to southern Canada in the north [11], [12]. Since the 1980s, the range of this species has expanded considerably, both to the west into New Mexico, Colorado, Wyoming, and South Dakota [13] as well as into the Great Lakes basin [14], [15].

During summer, *P. subflavus* roosts both in buildings [16] and in foliage [17]–[19]. Females may roost alone or in colonies, while males roost singly [12], [18]. In autumn, *P. subflavus* engage in swarming behavior, after which they hibernate in caves, abandoned mines and occasionally human-made structures [14], [15], [20]–[25]. There is little information about their movements among summering grounds, swarming sites, and hibernacula [26], but they are currently believed to be a short-distance regional migrant [12], [27], [28].

However, seasonal variation in abundance and sex ratios of *P. subflavus* has led some authors to speculate that individuals migrate farther distances than previously suspected [26], [29], [30] and that migratory behavior may vary between sexes [31], [32]. Further, recent studies have documented local increases in or an appearance of *P. subflavus* activity concurrent with increased activity of other latitudinal migrants during the autumn migration time period [33], [34]. Bats are frequently killed by wind turbines [26] and the species most susceptible to this type of mortality tend to engage in long-distance latitudinal migration [26], [35] and rely heavily on trees as roost sites. Within its range, *P. subflavus* is among the most frequently killed species around wind turbines and may account for up to 25% of total bat mortality [35], a much higher proportion than known regional migrants.

Stable hydrogen isotope analysis is now a common tool used to learn about the origin of migratory animals [36], [37]. There is a latitudinal pattern in the stable hydrogen isotope ratios of precipitation, with precipitation at more northern latitudes being increasingly depleted of deuterium compared to that at more southern latitudes. The stable hydrogen isotope composition of precipitation is recorded in the tissues of local animals. In the case of inert tissues such as fur and claw, stable hydrogen isotope composition is fixed at the time of formation and subsequent analyses of these tissues can provide information about the location of a migrant when the tissue was formed. Several authors have investigated tissue stable hydrogen isotope variability in bats across latitudes [38], and have used this tool to investigate altitudinal migration [39] and to describe annual migration in hoary bats (*Lasiurus cinereus*) [7].

The purpose of the current study was to investigate continental patterns in the annual movements of *P. subflavus* using stable hydrogen isotope analyses of fur samples. We predicted that the stable hydrogen isotope values of fur (δD_fur_) taken from animals collected between June and August, when the annual molt is believed to occur [7], would correlate closely with the latitude of capture [38], as well as the stable hydrogen isotope values of the predicted growing season precipitation (δD_precip_) at that location [7], [40]. Further, we predicted that the difference between δD_fur_ and δD_precip_ at the site of capture would be smallest during summer months (when fur is grown) and greater during winter (when the bats have migrated from the site of fur growth).

## Methods

### Sample collection and analysis

**Figure 1:**
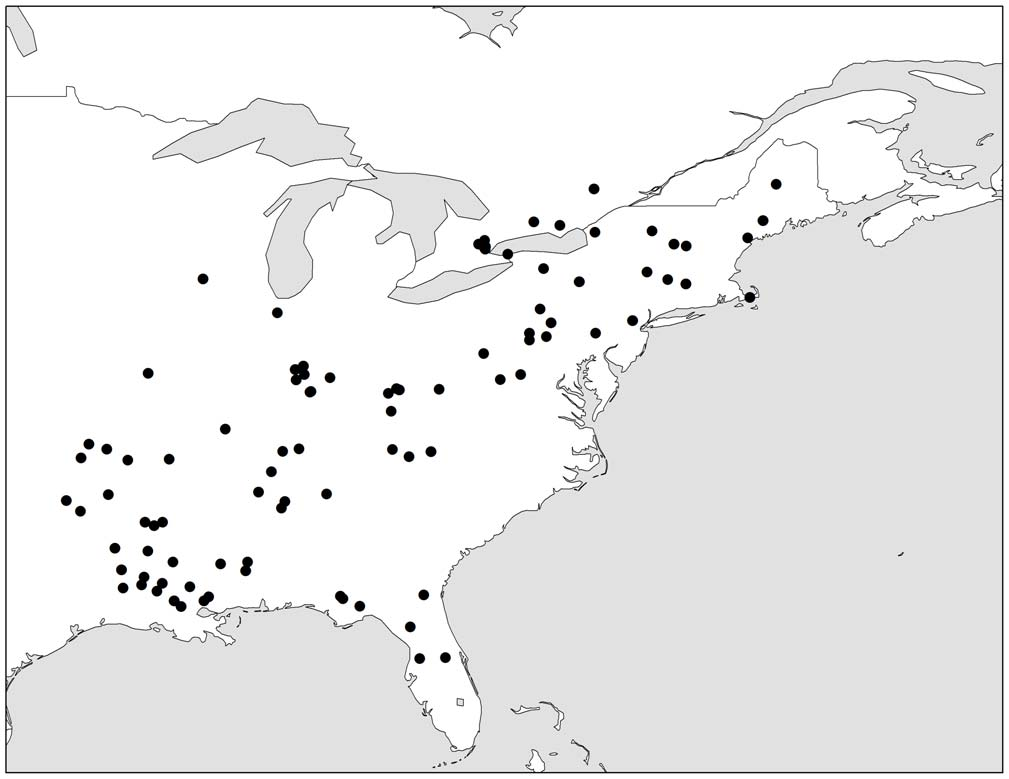
Tri-colored bat (*Perimyotis subflavus*) collection sites. Dorsal fur samples from 184 *Perimyotis subflavus* museum study skins were sampled from individuals collected across most of the species' range. Each black dot represents a collection location; multiple individuals were collected from some locations.

We obtained 184 fur samples taken from the lower dorsal region of *P. subflavus* study skins from four North American museum collections: Royal Ontario Museum (Toronto, ON), Louisiana State University Museum of Natural Science (Baton Rouge, LA), Harvard University Museum of Comparative Zoology (Cambridge, MA) and Cornell University Museum of Vertebrates (Ithaca, NY). Museum specimens were collected between 1878 and 1986 during all seasons and across most of the species' range (Figure 1; Table S1).

Fur samples were soaked overnight in a 2∶1 solution of chloroform∶methanol [41], rinsed the next day in the same solution and then dried in a fume hood for ≥48 hrs. Complex organic materials contain a fraction of hydrogen that is readily exchangeable with ambient vapor at room temperature. To correct for this uncontrolled exchange, we analyzed all samples alongside five in-house fur standards with known non-exchangeable δD values using a comparative equilibration approach [42]. Standards and samples were weighed into silver capsules (0.175 mg +/− 10 µg) with a 10% rate of duplication. After being weighed, samples and standards were left to equilibrate with laboratory air for a minimum of four days [43]. Samples were pyrolyzed using a High Temperature Conversion Elemental Analyzer (TC/EA) and analyzed using online continuous-flow isotope ratio mass spectrometry (IRMS). Results are expressed as parts per thousand (‰) relative to Vienna Standard Mean Ocean Water (VSMOW). Analytical precision, based on repeated analyses of fur from the same individual bat during each analysis, was less than 2‰ (1 standard deviation). The mean (± standard deviation) difference between duplicates of the same sample was 2±2‰.

### Data analysis

In some instances, GPS coordinates were available from individual museum databases for the locations of specimen collection. When this was not the case, coordinates were determined for the centroid of the county of collection using the United States Geological Survey Geographic Names Information System (http://geonames.usgs.gov/domestic/, accessed September 2010). Predicted growing season δD_precip_ values were determined for the collection locality of each specimen using the geospatial data available from waterisotopes.org [40], [44].

We conducted analyses of males and females separately and first plotted the difference between δD_fur_ and predicted growing season δD_precip_ at the location of capture (ΔD_fur-precip_) for individual bats against Julian date. Based on these results, we visually determined the time when bats were at their location of molt as the time period when ΔD_fur-precip_ was least variable (similar to the approach taken by [7]). This definition of the molting timeframe is an estimate based on proxy evidence and we did not directly observe fur replacement in this species. For the remainder of this manuscript, we refer to samples collected during the estimated molt period as having been collected during the “molt period”, and samples collected outside of the estimated molt period has having been collected during the “non-molt period”.

We then regressed the mean δD_fur_ values of bats collected during the molt period from each sampling location (number of bats from each location ranged from one to seven) against both collection latitude [38] and predicted growing season δD_precip_ values [40], [44], using linear and quadratic regressions for both predictors. We used the equations of the male and female δD_fur_/δD_precip_ regression lines to calculate predicted δD_fur_ values for each individual (based on the predicted growing season δD_precip_ value). In order to determine if there was isotopic evidence of latitudinal movement, and if so, if this behavior was more prevalent in some parts of the species' range than others, we plotted the difference between δD_fur_ and predicted δD_fur_ (ΔD_furactual-predict_) of all bats against Julian date and of all bats collected during the non-molt period against latitude.

We calculated the approximate origin of the individual bat that was farthest from its location of fur growth (greatest ΔD_furactual-predict_ value) by using the equation of the δD_fur_/δD_precip_ quadratic regression to calculate the predicted δD_precip_ value at its presumed location of fur growth. Substantial variation exists in the δD_fur_ values of bats collected from the same location during the molt period (which theoretically should be isotopically identical) [45], [46]. The expectation that one can estimate the δD_precip_ value at the location of fur growth based on the stable hydrogen isotope composition of one tissue sample is overly simplistic. Accordingly, to account for variation in molt period δD_fur_ values, we referred to the sex-specific range of ΔD_furactual-predict_ values that we recorded during the molt period and used the equation of the δD_fur_/δD_precip_ quadratic regression to calculate the δD_precip_ value associated with the individual bat's δD_fur_ value ±50% of the ΔD_furactual-predict_ variation. The results provide conservative estimates of the maximum and minimum δD_precip_ values at the presumed location of fur growth according to the available data on variation in ΔD_furactual-predict_ values during the molt period.

## Results

**Figure 2:**
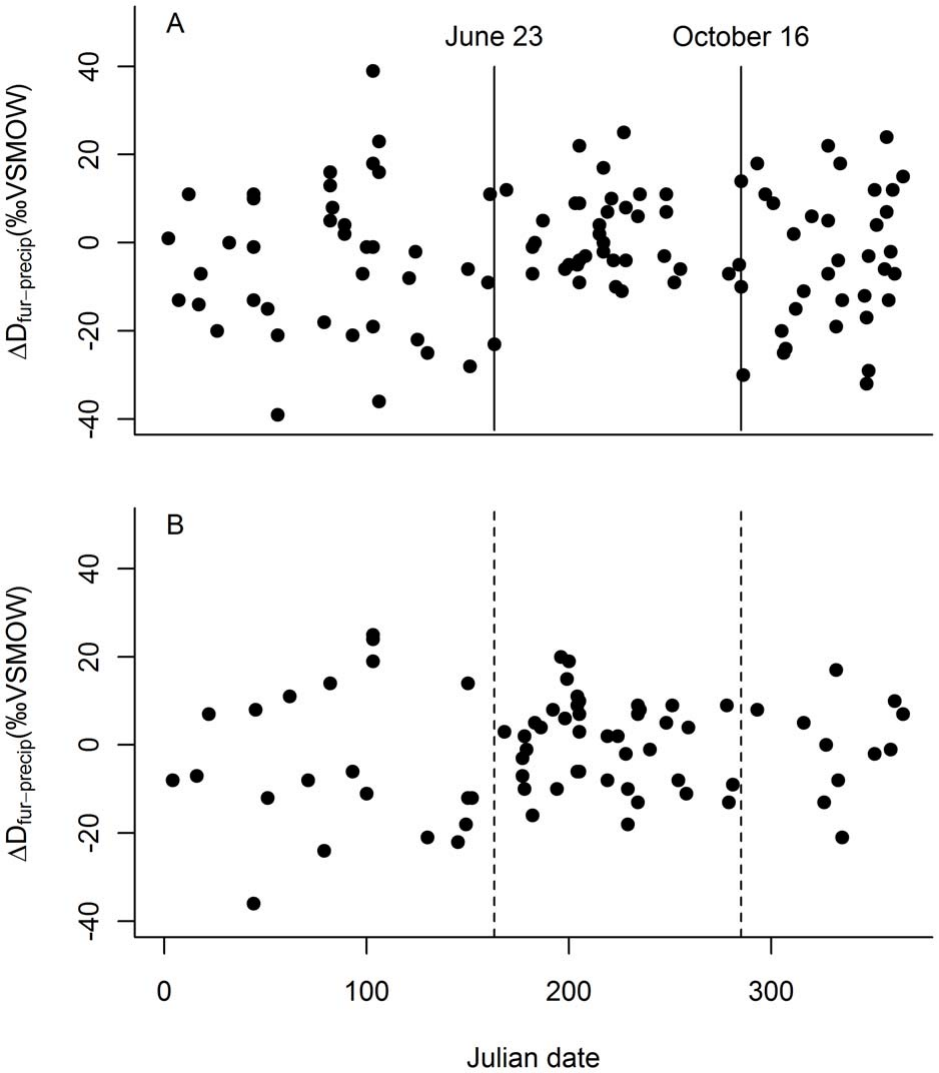
Molt period estimation. The difference between δD_fur_ and δD_precip_ (ΔD_fur-precip_) for males (panel A) was smallest between June 23 and October 16, as indicated by two vertical lines representing the estimated molt period (*n* = 111). There was no clear trend in female ΔD_fur-precip_ values across seasons (*n* = 73) (panel B), so the male estimated molt period was applied to females (dashed vertical lines).

Male ΔD_fur-precip_ values were least variable during the summer months, indicating that male *P. subflavus* molted between June 23 and October 16 (though a period for which we had no samples means that this end date may have been as early as September 9). For females, there was no clear period of low variability that would indicate a molt and then movement away from the molt location (Figure 2). Hereafter, we refer to the molt period as the time when the bat is at its location of fur replacement and define that time as being between June 23 and October 16. The molt period was indicated isotopically only by male bats and we make the explicit assumption that the female molt timing is identical to that of males.

**Figure 3:**
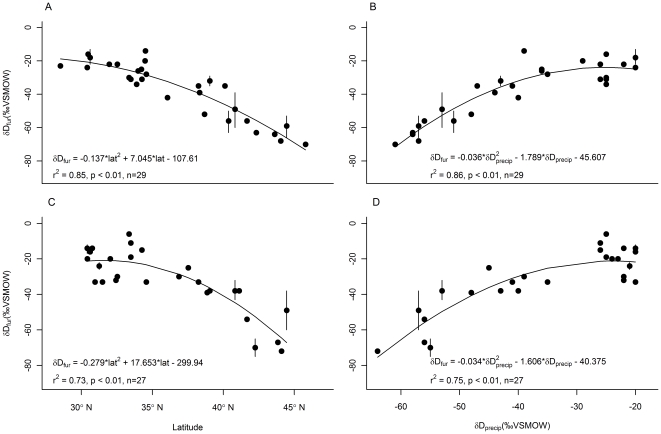
Regressions between molt period δD_fur_ and growing season δD_precip_ or latitude at the collection sites. The δD_fur_ values (site mean ± standard deviation) of males collected during the molt period varied significantly with latitude of capture (panel A) and predicted growing season δD_precip_ at the location of capture (panel B). The same was true of female molt period δD_fur_ values and latitude (panel C) and δD_precip_ (panel D). *n* = 1 to 7 individuals per site. Predicted growing season δD_precip_ values were obtained from waterisotopes.org [40], [44].

For each molt period δD_fur_/latitude and δD_fur_/δD_precip_ regression, the quadratic model was better than simple linear regression (indicated by higher adjusted *r* ^2^), and these are the relationships that we report. Male δD_fur_ values from the molt period correlated significantly with latitude of collection (*r* ^2^ = 0.85, *F* = 73.30, *df* = 28, *p*<0.01) and predicted growing season δD_precip_ at the collection site (*r* ^2^ = 0.86, *F* = 84.8, *df* = 28 *p*<0.01). Female molt period δD_fur_ values also correlated significantly with both variables (latitude – *r* ^2^ = 0.73, *F* = 35.4, *df* = 26, *p*<0.01; δD_precip_ – *r* ^2^ = 0.75, *F* = 39.3, *df* = 26, *p*<0.01) (Figure 3). Because latitude and δD_precip_ were almost equally effective at predicting molt period δD_fur_ values, we followed the advice of [47] and used the relationship between molt period δD_fur_ values and predicted growing season δD_precip_ to generate predicted δD_fur_ values for all bats.

**Figure 4:**
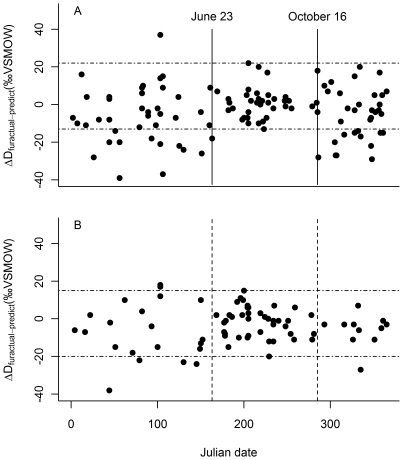
Identification of latitudinal migrants. The range of variation in molt period ΔD_furactual-predict_ values (where “molt period” refers to the estimated molt period shown in Figure 2) is indicated with horizontal dashed lines. Individuals with ΔD_furactual-predict_ values below the horizontal band likely did not grow their fur at their collection site, and are assumed to have migrated from a more northern location. Thirty-three percent of all males (*n* = 111) (panel A) and 16% of all females (*n* = 73) (panel B) collected during the non-molt period appeared to be south of their location of fur growth. One non-molt period male and two females appeared to be north of their location of fur growth.

Our initial plot of ΔD_fur-precip_ against Julian date (used to estimate molt period) implicitly assumed a linear relationship between molt period δD_fur_ values and predicted local growing season δD_precip_. Our data suggest that a quadratic curve better describes this relationship for both male and female bats. To correct for this, we used the sex-specific quadratic regression equations to calculate the predicted δD_fur_ value for each bat based on the predicted growing season δD_precip_ value at its location of capture [40], [44]. We re-did the initial plot to show ΔD_furactual-predict_ against Julian date for both male and female bats (Figure 4).

During the molt period, the difference between maximum and minimum ΔD_furactual-predict_ values was 35‰ for both males and females. During the non-molt period, the difference was 76‰ for males and 53‰ for females. Twenty-four of 73 males (32.9%) sampled during the non-molt period had ΔD_furactual-predict_ values that were more negative than any observed during the molt period, indicating that these individuals were captured south of their location of fur growth. One individual (1.4%) showed evidence of northward movement. Five of 32 females (15.6%) sampled during the non-molt period had ΔD_furactual-predict_ values indicative of a more northern location of fur growth, although in general, these values were not as negative as those observed among the male migrants. Two females captured during the non-molt period (6.3%) had ΔD_furactual-predict_ values that may have indicated a slight northward movement.

**Figure 5:**
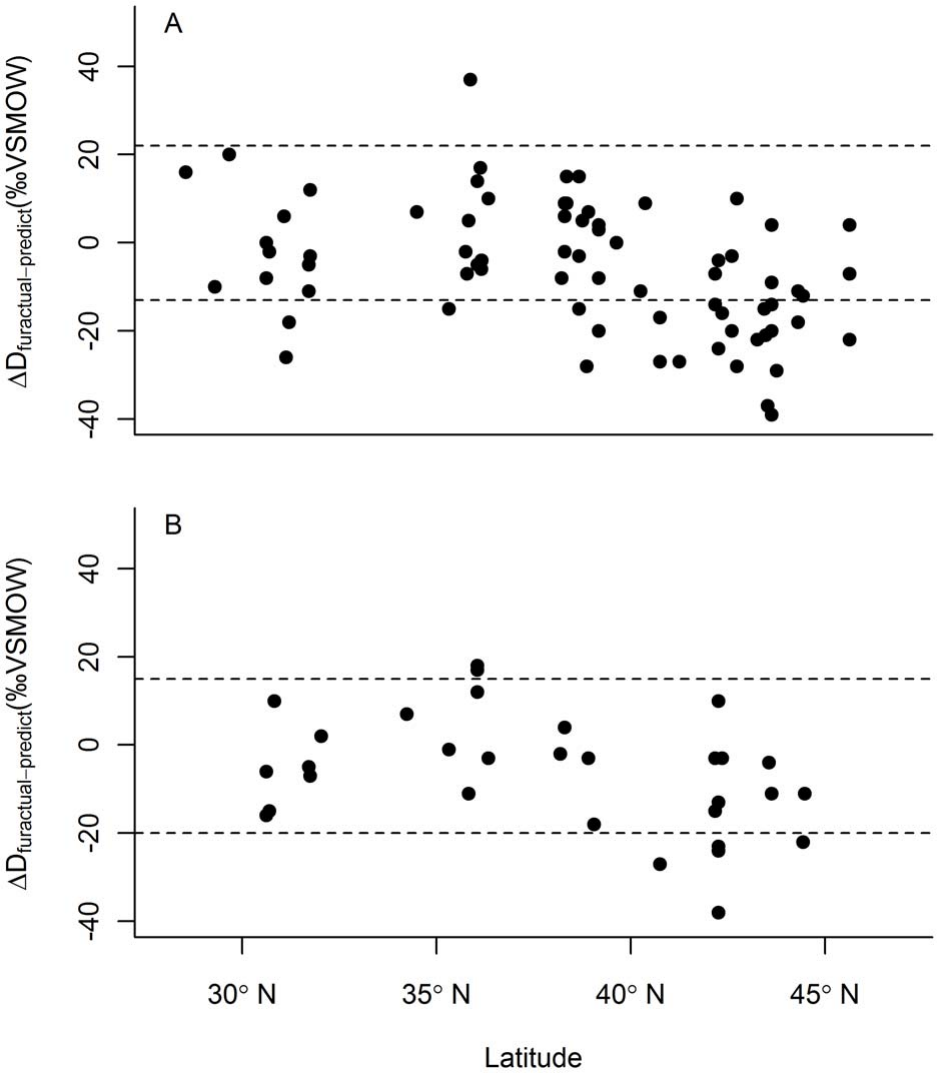
Latitudinal differences in migratory tendency. ΔD_furactual-predict_ values from the non-molt period were more negative than values from the molt period at both northern and southern latitudes for males (*n = *73) (panel A) and only at northern latitudes for females (*n = *32) (panel B), indicating southern migration from the latitude of fur growth by these individuals. The range of ΔD_furactual-predict_ values from the molt period is indicated by horizontal dashed lines.

For male bats, non-molt period ΔD_furactual-predict_ values were most negative at high (>40°) and low (<35°) latitudes and closest to the range of molt period values at mid-latitudes (Figure 5), suggesting that high and low latitude individuals engage in more substantial southern migrations than do mid-latitude males. The majority of the females with non-molt period ΔD_furactual-predict_ values indicating they were south of their location of fur growth were captured at the northern end of the species' range.

**Figure 6:**
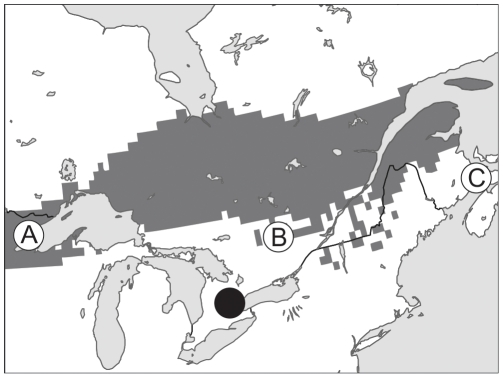
Estimated origin of the farthest migrant. The bat with the δD_furactual-predict_ value suggesting that it had migrated the farthest from its latitude of fur growth also had a δD_fur_ value indicating that it was from the most northern location (collection site indicated by black circle). It likely originated at a location where the mean growing season δD_precip_ composition was between −63 and −74‰ (the area shaded in grey). The lettered points indicate the existing most northern records of *Perimyotis subflavus* (A = [60]; B = [2]; C = [65]). Predicted growing season δD_precip_ values were obtained from waterisotopes.org [40], [44].

The bat that was most distinct in isotopic composition from the precipitation at its location of capture was a male collected from southwestern Ontario (43.62 decimal degrees N, −80.13 decimal degrees W). This bat also was one of two that had the most negative δD_fur_ composition (−93‰) of all bats sampled, indicating the most northern point of fur growth/origin. Based on an extrapolation of the relationship that we established between molt period δD_fur_ values and predicted growing season δD_precip_ at the location of capture, we predict that this individual bat grew its fur at a location with a mean predicted growing season δD_precip_ composition between −63‰ and −74‰ (Figure 6).

## Discussion

Our results indicate a fundamentally different picture of the annual migratory ecology of *P. subflavus* than has previously been assumed. *Perimyotis subflavus* visit swarming sites in autumn and hibernate during the winter [12], sharing many characteristics with other species that have been documented to engage in a regional radiation pattern of migration, moving from central hibernacula to summer maternity colonies (e.g., little brown bats (*Myotis lucifugus*) [1], [2]). However, the δD_fur_ composition of many of the bats collected during the non-molt period suggested that some individuals moved south of their region of fur growth and very few moved north. If *P. subflavus* only engages in regional, radiating migratory behavior typical of other species of bats that hibernate in caves, then we would expect to see evidence that equal numbers of bats migrate north and south during the non-molt period. Consequently we conclude that at least some *P. subflavus* of both sexes engage in the type of latitudinal migration that is more typically associated with hoary bats (*Lasiurus cinereus)*, eastern red bats *(Lasiurus borealis),* and silver-haired bats (*Lasionycteris noctivagans)* [6], and that this behavior is more common for males than for females.

### Sex-biased migration

Sex-biased migratory behavior is common among bats [28]. Typically, when sex-biases exist, females have a greater tendency to migrate than do males [6], [48]. The mechanisms driving female-biased migration have not been identified, but it has been hypothesized that the elevated energetic demands of pregnancy and lactation lead females to move to habitats where there is higher resource availability or access to roosts with better thermoregulatory properties [28]. Our data are in contrast to this trend, suggesting that males have a greater tendency to make long-distance north-south migrations than do females. One of the few examples of male-biased migration in bats was documented in the West African species *Myonycteris torquata* [49], and the authors hypothesized that females were limited in their movement by the costs of pregnancy and lactation while males were not similarly constrained and were able to track seasonal food resources. It is possible that temporal differences in reproductive-based energetic limitations may be a factor in causing the sex-biased migratory tendencies for which we see evidence in *P. subflavus.*

Male and female bats experience reproductive stress at different times of the year [50]. Females ovulate and become pregnant following emergence from hibernation and have increased energetic requirements throughout pregnancy and lactation in the early to mid-summer [51], [52]. Conversely, males experience reproductive costs in late summer during spermatogenesis [53], [54]. Variation in reproductive status has been linked to behavioral changes, such as increased food consumption [55], differential torpor use [56], [57], foraging bout duration [58] and home range size [59]. It is possible that reproductive demands upon emergence from hibernation preclude large-scale northern migration by females, whereas males are not subject to the same limitations.

### Effects of latitude on migratory tendency

All of the females and many of the males captured during the non-molt period with fur stable isotope compositions indicating that they were south of their location of fur growth, were collected from the northern part of the *P. subflavus* range. In some cases, δD_fur_ values from the non-molt period indicated that the fur had been grown at least at the northern extent of the known range for the species [2], [12], [60], [65], and perhaps even farther north than these locations. We suggest that some *P. subflavus* summering at the extreme northern edge of the range may migrate south (although still remaining in the northern portion of the known species range) to hibernate at sites where winters are shorter and their probability of survival is higher [61].

Previous research [62] suggests that while winter length is a factor in survivorship of hibernating little brown bats (*Myotis lucifugus*), a more important factor is the extent to which the bats hibernate in clusters. Hibernating in clusters decreases the amount of energy lost during normothermic bouts within the hibernation period [63] and as long as bats are hibernating in clusters, the impact of varying winter length on survivorship is negligible [62]. However, the hibernation ecology of *P. subflavus* differs from that of many other species; it is well documented hibernating singly or occasionally in small clusters, but not frequently in large clusters [12], [15], [24], [64]–[67]. We suggest that hibernating singly makes *P. subflavus* more susceptible to the longer winters at northern hibernacula than clustering species; resulting in an increased probability of survival for individuals who migrate south to hibernate.

### Bat residency and the molt period

Interpretation of our results relies heavily on the timing and location of bat fur growth, so it is appropriate to consider molting patterns of *P. subflavus.* Previous studies of molting patterns of temperate bats suggest that molt usually occurs once annually between mid-June and September [7], [68], [69], similar to the period that we have defined for male *P. subflavus.* However, it is important to remember that our isotopically estimated molt period does not describe the period of actual hair replacement, but only the period when the bat remains a resident at its location of fur growth; *i.e.*, fur replacement happens at some point during the isotopically defined “molt period”. Currently we know of no data documenting inter-annual consistency in resident bat δD_fur_ values, and *P. subflavus* philopatry is poorly understood, although there is evidence that females return to the same summer roosting sites each year [16], [70]. It is unclear if the initial date of the molt period (June 23) is actually indicative of the beginning of fur replacement or just the date when all sampled bats had returned to regular summering grounds and hence had fur grown the previous year that was still isotopically indicative of their location of capture.

Our earliest estimate for when bats may have begun to leave their location of fur growth is September 9. This date is a much later start to the autumn migratory movement than has previously been recorded for *P. subflavus* specifically, and for other North American bat species in general. In Missouri, subadult *P. subflavus* started arriving at swarming sites on August 5 [29] and studies of other swarming and hibernating species indicate that male bats begin to arrive at swarming sites as early as August 1 [2]. Our stable isotope results may indicate a tendency for bats to only engage in long distance southern migration in mid to late fall, but we think that the late migration date that we detected is more likely an artifact of sampling bias. Logistically, the easiest places to capture and collect a colonial species such as *P. subflavus* are summer colonies and winter hibernacula. Little is known about the mobility and roosting habits of these bats during the late summer and fall when they engage in swarming behavior, making them challenging to reliably locate. Minimal collection data exists for the specimens used in this study, so we do not know the circumstance in which most were collected. If the late summer bats included in this study were collected from known summer colonies, then they may represent a minority of bats that remained at their summer location into the late summer and early fall and may not be representative of the majority of *P. subflavus*. A follow-up study using samples collected from bats captured at swarming sites during the fall season may clarify this point.

Variation in female fur isotopic compositions was similar throughout the year, so we could not identify a time period for the female molt. Throughout this study, we have applied the male isotopically defined molt period to both sexes, which is a necessary but potentially problematic approach. Other authors [7], [68], [71] reported that male and female molt patterns may vary in timing, likely as a function of the increased energetic demands faced by females during reproduction. Male *Tadarida brasiliensis* and *Myotis velifer* molted before female conspecifics [68] and female *Miniopterus schreibersii* ceased molting during lactation [71]. Delayed molt timing in females could translate to decreased isotopic migration detectability. If females are still molting as they begin to migrate, then the stable isotope signature of the food and water that they drink at a range of locations will be integrated into their fur. The δD_fur_ values would not be indicative of one location of fur growth, but the average of a range of locations.

### Summary - Perimyotis subflavus as a partial and differential latitudinal migrant

We found that the δD_fur_ values of *P. subflavus* collected during the molt period are a good predictor of both the latitude and the predicted local growing season δD_precip_ of the collection location and so can be used as an indicator of bat movement away from that location. Stable isotope evidence suggested that some bats of both sexes underwent a southern fall migration during the non-molt period and that this behavior was more prevalent in males than in females. The majority of individuals for which isotope values suggested a latitudinal migration (both sexes) were captured in the northern portion of the species' range. Some non-molt period males from the southern portion of the range also showed evidence of southern migration.

It is important to note that migration is a characteristic of individuals and not of populations or species [28], [72]. Migratory behavior varies greatly among individuals based on a variety of intrinsic and extrinsic factors. Both partial migration (movement of some members of a species, but not all) and differential migration (sex or age cohorts exhibiting different migratory patterns) [73] are common in bat species [28]. Our data suggest that *P. subflavus* can be described as both a partial migrant and differential migrant, as stable isotope results provide evidence of differences in migratory behavior between sexes and among latitudes. Though some individuals may undertake regional radiation migrations as previously suggested, our evidence suggests that latitudinal migration is a strategy undertaken by a large proportion of individuals.

## Supporting Information

Table S1 **Collection and stable isotope data for all specimens.** Each bat is identified with a museum-specific abbreviation (CU - Cornell University Museum of Vertebrates, Ithaca, NY; LSUMZ - Louisiana State University Museum of Natural Science, Baton Rouge, LA; MCZ – Harvard University Museum of Comparative Zoology, Cambridge, MA; ROM - Royal Ontario Museum, Toronto, ON) and its associated catalog number. Collection coordinates for each specimen are included in decimal degrees. For some specimens, exact collection coordinates were available. For specimens where exact collection data were not available, coordinates for the centroid of the county of collection were used and reported below (obtained from the United States Geological Survey Geographic Names Information System (http://geonames.usgs.gov/domestic/, accessed September 2010). Predicted growing season δD_precip_ values were obtained from waterisotopes.org [40], [44]. All stable isotope data are reported in ‰ relative to VSMOW. F = female; M = male.
(DOC)
