# Supplementary material for: Evidence of Latitudinal Migration in Tri-colored Bats, Perimyotis subflavus
Source: PLoS One. 2012 Feb 22;7(2):e31419. doi: 10.1371/journal.pone.0031419 (PMC3284490; doi:10.1371/journal.pone.0031419)
Supplement: Table S1 — Collection and stable isotope data for all specimens. Each bat is identified with a museum-specific abbreviation (CU - Cornell University Museum of Vertebrates, Ithaca, NY; LSUMZ - Louisiana State University Museum of Natural Science, Baton Rouge, LA; MCZ – Harvard University Museum of Comparative Zoology, Cambridge, MA; ROM - Royal Ontario Museum, Toronto, ON) and its associated catalog number. Collection coordinates for each specimen are included in decimal degrees. For some specimens, exact collection coordinates were available. For specimens where exact collection data were not available, coordinates for the centroid of the county of collection were used and reported below (obtained from the United States Geological Survey Geographic Names Information System (http://geonames.usgs.gov/domestic/, accessed September 2010). Predicted growing season δDprecip values were obtained from waterisotopes.org [40], [44]. All stable isotope data are reported in ‰ relative to VSMOW. F = female; M = male. (DOC) [file pone.0031419.s001.doc]

Table S1.

| **Bat** | **State/Province** | **Sex** | **Year** | **Julian date** | **δD_fur_** | **Latitude** | **Longitude** | **δD_precip_** |
| --- | --- | --- | --- | --- | --- | --- | --- | --- |
| LSUMZ 18633 | Alabama | F | 1974 | 332 | -19 | 34.24 | -87.20 | -36 |
| LSUMZ 11582 | Alabama | M | 1966 | 221 | -26 | 34.00 | -87.32 | -36 |
| LSUMZ 18632 | Alabama | M | 1974 | 235 | -25 | 34.24 | -87.20 | -36 |
| LSUMZ 761 | Alabama | M | 1935 | 227 | -14 | 34.52 | -85.67 | -39 |
| LSUMZ 11579 | Arkansas | F | 1966 | 200 | -6 | 33.36 | -91.96 | -25 |
| LSUMZ 11574 | Arkansas | F | 1966 | 198 | -19 | 33.49 | -92.29 | -25 |
| LSUMZ 11571 | Arkansas | F | 1966 | 199 | -11 | 33.49 | -91.65 | -26 |
| MCZ 29938 | Arkansas | F | 1933 | 293 | -30 | 36.33 | -94.33 | -38 |
| LSUMZ 11578 | Arkansas | M | 1966 | 200 | -30 | 33.36 | -91.96 | -25 |
| LSUMZ 11576 | Arkansas | M | 1966 | 198 | -31 | 33.49 | -92.29 | -25 |
| LSUMZ 20389 | Arkansas | M | 1976 | 297 | -18 | 34.49 | -93.63 | -29 |
| LSUMZ 11570 | Arkansas | M | 1966 | 203 | -20 | 34.49 | -93.63 | -29 |
| LSUMZ 19710 | Arkansas | M | 1973 | 320 | -30 | 35.75 | -92.92 | -36 |
| LSUMZ 15179 | Arkansas | M | 1969 | 2 | -34 | 35.78 | -91.41 | -35 |
| ROM 999999 | Arkansas | M | 1934 | 89 | -35 | 36.15 | -93.68 | -37 |
| ROM 3412090003 | Arkansas | M | 1934 | 89 | -33 | 36.15 | -93.68 | -37 |
| MCZ 29937 | Arkansas | M | 1933 | 293 | -20 | 36.33 | -94.33 | -38 |
| ROM 76294 | Florida | F | 1959 | 186 | -20 | 30.42 | -84.47 | -24 |
| LSUMZ 11142 | Florida | F | 1966 | 100 | -36 | 30.70 | -85.08 | -25 |
| MCZ BANGS-5598 | Florida | M | 1894 | 247 | -23 | 28.52 | -82.28 | -20 |
| ROM 76292 | Florida | M | 1951 | 12 | -9 | 28.55 | -81.35 | -20 |
| ROM 76293 | Florida | M | 1963 | 334 | -4 | 29.67 | -82.63 | -22 |
| LSUMZ 11141 | Florida | M | 1966 | 100 | -26 | 30.70 | -85.08 | -25 |
| ROM 76295 | Florida | M | 1959 | 205 | -16 | 30.79 | -85.17 | -25 |
| CU 163 | Georgia | F | 1921 | 150 | -11 | 30.83 | -82.13 | -25 |
| MCZ 46625 | Illinois | F | 1947 | 316 | -42 | 42.35 | -90.18 | -47 |
| MCZ 46626 | Illinois | M | 1947 | 316 | -58 | 42.35 | -90.18 | -47 |
| ROM 76298 | Indiana | F | 1974 | 234 | -34 | 38.25 | -86.25 | -41 |
| ROM 76304 | Indiana | F | 1974 | 234 | -32 | 38.25 | -86.25 | -41 |
| ROM 76297 | Indiana | F | 1973 | 71 | -51 | 39.05 | -86.82 | -43 |
| ROM 76296 | Indiana | F | 1974 | 248 | -38 | 39.05 | -86.82 | -43 |
| ROM 45577 | Indiana | F | 1965 | 178 | -38 | 41.12 | -87.47 | -40 |
| ROM 45578 | Indiana | M | 1968 | 32 | -41 | 38.22 | -86.28 | -41 |
| ROM 76299 | Indiana | M | 1974 | 234 | -35 | 38.25 | -86.25 | -41 |
| ROM 45579 | Indiana | M | 1967 | 328 | -48 | 38.67 | -86.78 | -41 |
| ROM 45585 | Indiana | M | 1967 | 328 | -19 | 38.67 | -86.78 | -41 |
| ROM 45591 | Indiana | M | 1967 | 328 | -36 | 38.67 | -86.78 | -41 |
| ROM 45621 | Indiana | M | 1956 | 360 | -31 | 38.75 | -85.55 | -43 |
| ROM 45580 | Indiana | M | 1967 | 26 | -62 | 38.86 | -86.49 | -42 |
| ROM 76300 | Indiana | M | 1974 | 248 | -36 | 39.05 | -86.82 | -43 |
| ROM 76301 | Indiana | M | 1974 | 248 | -32 | 39.05 | -86.82 | -43 |
| ROM 45581 | Indiana | M | 1966 | 44 | -56 | 39.17 | -86.52 | -43 |
| ROM 45582 | Indiana | M | 1966 | 44 | -32 | 39.17 | -86.52 | -43 |
| ROM 45583 | Indiana | M | 1966 | 44 | -33 | 39.17 | -86.52 | -43 |
| ROM 45584 | Indiana | M | 1966 | 44 | -44 | 39.17 | -86.52 | -43 |
| CU 881 | Kentucky | F | 1925 | 196 | -25 | 37.53 | -83.32 | -45 |
| CU 10375 | Kentucky | F | 1939 | 45 | -35 | 38.18 | -83.43 | -43 |
| MCZ 46624 | Kentucky | F | 1940 | 82 | -29 | 38.30 | -83.03 | -43 |
| MCZ 40976 | Kentucky | M | 1940 | 82 | -38 | 38.30 | -83.03 | -43 |
| MCZ 40977 | Kentucky | M | 1940 | 82 | -27 | 38.30 | -83.03 | -43 |
| MCZ 46623 | Kentucky | M | 1940 | 82 | -30 | 38.30 | -83.03 | -43 |
| ROM 22955 | Kentucky | M | 1950 | 106 | -27 | 38.35 | -83.13 | -43 |
| ROM 22956 | Kentucky | M | 1950 | 106 | -20 | 38.35 | -83.13 | -43 |
| LSUMZ 11599 | Louisiana | F | 1966 | 192 | -12 | 30.41 | -90.98 | -20 |
| LSUMZ 11600 | Louisiana | F | 1966 | 228 | -22 | 30.41 | -90.98 | -20 |
| LSUMZ 3321 | Louisiana | F | 1949 | 4 | -28 | 30.62 | -91.23 | -20 |
| LSUMZ 11138 | Louisiana | F | 1966 | 149 | -38 | 30.62 | -91.23 | -20 |
| LSUMZ 34147 | Louisiana | F | 1986 | 333 | -28 | 30.62 | -90.14 | -20 |
| LSUMZ 6776 | Louisiana | F | 1951 | 259 | -16 | 30.62 | -91.23 | -20 |
| LSUMZ 3671 | Louisiana | F | 1949 | 235 | -14 | 30.77 | -89.97 | -22 |
| LSUMZ 29877 | Louisiana | F | 1972 | 279 | -33 | 30.97 | -91.86 | -20 |
| LSUMZ 11597 | Louisiana | F | 1966 | 179 | -22 | 31.26 | -91.66 | -21 |
| LSUMZ 11598 | Louisiana | F | 1966 | 281 | -30 | 31.26 | -91.66 | -21 |
| LSUMZ 25413 | Louisiana | F | 1981 | 234 | -33 | 31.49 | -92.32 | -20 |
| LSUMZ 1973 | Louisiana | F | 1941 | 16 | -28 | 31.75 | -93.14 | -21 |
| LSUMZ 6479 | Louisiana | F | 1954 | 168 | -20 | 32.04 | -91.27 | -23 |
| LSUMZ 11595 | Louisiana | F | 1966 | 194 | -32 | 32.44 | -92.19 | -22 |
| LSUMZ 10551 | Louisiana | F | 1965 | 254 | -30 | 32.54 | -93.39 | -22 |
| LSUMZ 26732 | Louisiana | M | 1983 | 7 | -34 | 29.30 | 89.48 | -21 |
| LSUMZ 11601 | Louisiana | M | 1966 | 228 | -24 | 30.41 | -90.98 | -20 |
| LSUMZ 34146 | Louisiana | M | 1986 | 333 | -24 | 30.62 | -90.14 | -20 |
| LSUMZ 3320 | Louisiana | M | 1948 | 346 | -32 | 30.62 | -91.23 | -20 |
| LSUMZ 9299 | Louisiana | M | 1963 | 208 | -23 | 30.62 | -91.23 | -20 |
| LSUMZ 9300 | Louisiana | M | 1963 | 215 | -18 | 30.62 | -91.23 | -20 |
| LSUMZ 2494 | Louisiana | M | 1947 | 285 | -6 | 30.62 | -91.23 | -20 |
| LSUMZ 25224 | Louisiana | M | 1981 | 311 | -18 | 31.08 | -93.09 | -20 |
| LSUMZ 10552 | Louisiana | M | 1965 | 163 | -43 | 31.20 | -92.42 | -20 |
| LSUMZ 1423 | Louisiana | M | 1939 | 301 | -12 | 31.75 | -93.14 | -21 |
| LSUMZ 1109 | Louisiana | M | 1938 | 356 | -27 | 31.75 | -93.14 | -21 |
| LSUMZ 11594 | Louisiana | M | 1966 | 183 | -22 | 32.54 | -93.39 | -22 |
| MCZ 55622 | Maine | F | 1903 | 258 | -67 | 43.85 | -70.33 | -56 |
| MCZ 55627 | Maine | F | 1912 | 152 | -69 | 44.47 | -69.77 | -57 |
| MCZ 55624 | Maine | F | 1915 | 177 | -64 | 44.47 | -69.77 | -57 |
| MCZ 55625 | Maine | F | 1915 | 177 | -60 | 44.47 | -69.77 | -57 |
| MCZ 55630 | Maine | F | 1914 | 178 | -67 | 44.47 | -69.77 | -57 |
| MCZ 55628 | Maine | F | 1913 | 182 | -73 | 44.47 | -69.77 | -57 |
| MCZ 55629 | Maine | F | 1909 | 240 | -58 | 44.47 | -69.77 | -57 |
| MCZ 55626 | Maine | M | 1916 | 182 | -58 | 44.47 | -69.77 | -57 |
| MCZ 55631 | Maine | M | 1914 | 182 | -64 | 44.47 | -69.77 | -57 |
| MCZ 55633 | Maine | M | 1916 | 255 | -63 | 44.47 | -69.77 | -57 |
| MCZ 55623 | Maine | M | 1913 | 252 | -70 | 45.80 | -69.30 | -61 |
| MCZ BANGS-5596 | Massachusetts | F | 1878 | 224 | -54 | 41.67 | -70.25 | -56 |
| MCZ 34654 | Massachusetts | F | 1937 | 51 | -66 | 42.17 | -72.58 | -54 |
| MCZ 34580 | Massachusetts | F | 1936 | 327 | -54 | 42.17 | -72.58 | -54 |
| CU 7869 | Massachusetts | M | 1953 | 217 | -39 | 41.67 | -70.25 | -56 |
| CU 7870 | Massachusetts | M | 1953 | 217 | -56 | 41.67 | -70.25 | -56 |
| MCZ 34653 | Massachusetts | M | 1937 | 51 | -69 | 42.17 | -72.58 | -54 |
| MCZ 37489 | Massachusetts | M | 1937 | 121 | -62 | 42.17 | -72.58 | -54 |
| MCZ 34553 | Massachusetts | M | 1936 | 284 | -63 | 42.33 | -73.25 | -58 |
| LSUMZ 8727 | Mississippi | F | 1960 | 359 | -27 | 31.71 | -88.63 | -26 |
| LSUMZ 10956 | Mississippi | F | 1966 | 22 | -20 | 32.03 | -88.55 | -27 |
| LSUMZ 11589 | Mississippi | F | 1966 | 219 | -33 | 34.58 | -88.15 | -35 |
| LSUMZ 11591 | Mississippi | M | 1966 | 151 | -50 | 31.13 | -90.66 | -22 |
| LSUMZ 11593 | Mississippi | M | 1966 | 160 | -35 | 31.71 | -88.63 | -26 |
| LSUMZ 8728 | Mississippi | M | 1960 | 359 | -28 | 31.71 | -88.63 | -26 |
| LSUMZ 11590 | Mississippi | M | 1966 | 215 | -22 | 31.97 | -89.54 | -26 |
| LSUMZ 11588 | Mississippi | M | 1966 | 219 | -28 | 34.58 | -88.15 | -35 |
| LSUMZ 11561 | Missouri | F | 1966 | 251 | -30 | 36.88 | -89.36 | -39 |
| LSUMZ 6155 | Missouri | F | 1950 | 365 | -33 | 38.91 | -92.18 | -40 |
| LSUMZ 6156 | Missouri | M | 1950 | 365 | -25 | 38.91 | -92.18 | -40 |
| CU 7836 | New Jersey | F | 1940 | 183 | -48 | 40.83 | -74.53 | -53 |
| CU 7837 | New Jersey | F | 1940 | 204 | -44 | 40.83 | -74.53 | -53 |
| CU 7838 | New Jersey | F | 1940 | 204 | -59 | 40.83 | -74.53 | -53 |
| CU 7833 | New Jersey | F | 1940 | 205 | -46 | 40.83 | -74.53 | -53 |
| CU 7835 | New Jersey | F | 1940 | 205 | -50 | 40.83 | -74.53 | -53 |
| CU 7839 | New Jersey | F | 1940 | 205 | -43 | 40.83 | -74.53 | -53 |
| CU 7841 | New Jersey | F | 1940 | 205 | -59 | 40.83 | -74.53 | -53 |
| CU 7834 | New Jersey | M | 1940 | 205 | -31 | 40.83 | -74.53 | -53 |
| CU 7840 | New Jersey | M | 1940 | 205 | -57 | 40.83 | -74.53 | -53 |
| CU 822 | New York | F | 1926 | 44 | -91 | 42.25 | -76.47 | -55 |
| CU 7831 | New York | F | 1929 | 62 | -44 | 42.25 | -76.47 | -55 |
| CU 880 | New York | F | 1924 | 130 | -76 | 42.25 | -76.47 | -55 |
| CU 7827 | New York | F | 1907 | 145 | -77 | 42.25 | -76.47 | -55 |
| CU 702 | New York | F | 1924 | 150 | -67 | 42.25 | -76.47 | -55 |
| CU 9599 | New York | F | 1953 | 229 | -73 | 42.25 | -76.47 | -55 |
| CU 9600 | New York | F | 1953 | 229 | -65 | 42.25 | -76.47 | -55 |
| MCZ 41941 | New York | F | 1896 | 219 | -72 | 44.10 | -73.82 | -64 |
| CU 878 | New York | M | 1924 | 130 | -80 | 42.25 | -76.47 | -55 |
| CU 701 | New York | M | 1924 | 150 | -61 | 42.25 | -76.47 | -55 |
| MCZ 48533 | New York | M | 1941 | 305 | -74 | 42.60 | -74.00 | -54 |
| MCZ 48535 | New York | M | 1949 | 348 | -57 | 42.60 | -74.00 | -54 |
| CU 19679 | New York | M | 1973 | 83 | -47 | 42.73 | -77.77 | -55 |
| CU 12875 | New York | M | 1967 | 286 | -85 | 42.73 | -77.77 | -55 |
| CU 7740 | New York | M | 1953 | 226 | -68 | 44.05 | -75.90 | -57 |
| ROM 22950 | North Carolina | F | 1950 | 103 | -24 | 36.05 | -81.87 | -49 |
| ROM 22951 | North Carolina | F | 1950 | 103 | -25 | 36.05 | -81.87 | -49 |
| ROM 22954 | North Carolina | F | 1950 | 103 | -30 | 36.05 | -81.87 | -49 |
| MCZ 11399 | North Carolina | M | 1912 | 103 | -13 | 35.87 | -82.67 | -52 |
| ROM 22952 | North Carolina | M | 1950 | 103 | -31 | 36.05 | -81.87 | -49 |
| ROM 22953 | North Carolina | M | 1950 | 103 | -50 | 36.05 | -81.87 | -49 |
| LSUMZ 11562 | Oklahoma | F | 1966 | 204 | -15 | 34.27 | -95.16 | -26 |
| ROM 19677 | Oklahoma | F | 1948 | 351 | -35 | 35.83 | -94.62 | -33 |
| LSUMZ 11566 | Oklahoma | M | 1966 | 205 | -34 | 33.89 | -94.64 | -25 |
| LSUMZ 11563 | Oklahoma | M | 1966 | 204 | -31 | 34.27 | -95.16 | -26 |
| ROM 19676 | Oklahoma | M | 1948 | 351 | -21 | 35.83 | -94.62 | -33 |
| ROM 83037 | Ontario | F | 1966 | 79 | -82 | 44.43 | -78.13 | -58 |
| ROM 3305150002 | Ontario | M | 1933 | 125 | -75 | 43.25 | -79.07 | -53 |
| ROM 33660 | Ontario | M | 1957 | 358 | -65 | 43.43 | -79.90 | -52 |
| ROM 21809 | Ontario | M | 1952 | 103 | -71 | 43.47 | -79.92 | -52 |
| ROM 20948 | Ontario | M | 1951 | 106 | -88 | 43.52 | -79.88 | -52 |
| ROM 14338 | Ontario | M | 1940 | 56 | -93 | 43.62 | -80.13 | -54 |
| ROM 14339 | Ontario | M | 1940 | 56 | -75 | 43.62 | -80.13 | -54 |
| ROM 22498 | Ontario | M | 1952 | 348 | -83 | 43.75 | -79.92 | -54 |
| ROM 14850 | Ontario | M | 1941 | 17 | -70 | 44.30 | -77.18 | -56 |
| ROM 16062 | Ontario | M | 1942 | 93 | -77 | 44.30 | -77.18 | -56 |
| ROM 83035 | Ontario | M | 1966 | 79 | -76 | 44.43 | -78.13 | -58 |
| CU 3945 | Pennsylvania | F | 1940 | 335 | -71 | 40.75 | -77.50 | -50 |
| ROM 24686 | Pennsylvania | M | 1932 | 169 | -35 | 40.12 | -78.28 | -47 |
| CU 7298 | Pennsylvania | M | 1947 | 98 | -56 | 40.25 | -77.67 | -49 |
| ROM 24685 | Pennsylvania | M | 1932 | 161 | -40 | 40.37 | -78.28 | -51 |
| ROM 24683 | Pennsylvania | M | 1931 | 223 | -61 | 40.37 | -75.88 | -51 |
| ROM 24684 | Pennsylvania | M | 1931 | 228 | -43 | 40.37 | -78.28 | -51 |
| MCZ 48534 | Pennsylvania | M | 1941 | 307 | -74 | 40.75 | -77.50 | -50 |
| CU 3942 | Pennsylvania | M | 1940 | 335 | -63 | 40.75 | -77.50 | -50 |
| CU 725 | Pennsylvania | M | 1924 | 306 | -76 | 41.25 | -77.90 | -51 |
| ROM 14849 | Quebec | M | 1941 | 18 | -68 | 45.63 | -75.93 | -61 |
| ROM 22948 | Quebec | M | 1946 | 347 | -78 | 45.63 | -75.93 | -61 |
| ROM 22949 | Quebec | M | 1946 | 347 | -93 | 45.63 | -75.93 | -61 |
| LSUMZ 3669 | Tennessee | F | 1949 | 361 | -29 | 35.32 | -87.69 | -39 |
| LSUMZ 3670 | Tennessee | M | 1949 | 361 | -46 | 35.32 | -87.69 | -39 |
| LSUMZ 19498 | Tennessee | M | 1950 | 217 | -42 | 36.07 | -87.28 | -40 |
| MCZ 11398 | Tennessee | M | 1911 | 357 | -20 | 36.13 | -83.28 | -44 |
| LSUMZ 19500 | Tennessee | M | 1949 | 352 | -36 | 36.16 | -86.68 | -40 |
| MCZ 37490 | Vermont | F | 1935 | 93 | -64 | 43.55 | -72.57 | -58 |
| MCZ 59115 | Vermont | F | 1914 | 326 | -71 | 43.62 | -73.02 | -58 |
| MCZ 59113 | Vermont | M | 1913 | 124 | -60 | 43.62 | -73.02 | -58 |
| MCZ 59117 | Vermont | M | 1934 | 312 | -73 | 43.62 | -73.02 | -58 |
| MCZ 59116 | Vermont | M | 1915 | 332 | -77 | 43.62 | -73.02 | -58 |
| MCZ 59114 | Vermont | M | 1914 | 279 | -65 | 43.62 | -73.02 | -58 |
| MCZ 35435 | Vermont | M | 1937 | 285 | -68 | 43.62 | -73.02 | -58 |
| CU 591 | Virginia | F | 1923 | 278 | -39 | 38.87 | -78.60 | -48 |
| CU 1082 | West Virginia | M | 1931 | 187 | -39 | 38.33 | -81.57 | -44 |
| CU 1078 | West Virginia | M | 1931 | 222 | -52 | 38.68 | -79.35 | -48 |
| ROM 10388 | West Virginia | M | 1936 | 357 | -38 | 39.63 | -79.95 | -45 |
